# Comparison of Ureteroscopic Pneumatic Lithotripsy and Extracorporeal Shock Wave Lithotripsy for Proximal Ureteral Calculi

**DOI:** 10.7759/cureus.7840

**Published:** 2020-04-26

**Authors:** Muhammad Fazal Ur Rehman, Muhammad Adnan, Ali Hassan, Fawad Humayun Akhtar, Naseem Javed, Farman Ali

**Affiliations:** 1 Urology, Bakhtawar Amin Medical and Dental College, Multan, PAK; 2 Urology, University of Lahore Teaching Hospital, Lahore, PAK; 3 Urology, Children Hospital and Institute of Child Health, Lahore, PAK; 4 Paediatric Urology, Children Hospital and Institute of Child Health, Lahore, PAK; 5 Internal Medicine, District Headquarter Hospital, Khanewal, PAK

**Keywords:** complications, eswl, stone-free rate, proximal ureter stone, urs

## Abstract

Objective

The goal of this study was to compare the effectiveness and complications of ureteroscopic pneumatic lithotripsy (URS) and extracorporeal shock wave lithotripsy (SWL) in the management of patients with proximal ureteral stones.

Methods

In this trial, 150 patients presenting with proximal ureteral stones at the Department of Urology of Nishter Hospital Multan from November 2018 to November 2019 were allocated 1:1 to undergo URS or SWL. The presence of stone fragments <4 mm on follow-up was regarded as being stone free. The study outcomes included stone-free rates after first, second, and third treatment sessions and stone retropulsion into the kidneys.

Results

A total of 75 patients each underwent URS and SWL. The mean procedure times for SWL and URS were 61.61± 3.21 and 85.01±6.75 minutes, respectively (P=0.000), and the mean numbers of procedures were 1.51±0.49 and 1.01±0.42, respectively (P=0.000). Stone-free rates after the first, second, and third sessions of SWL were 64%, 77.3%, and 94.7%, respectively, whereas stone-free rates after the first, second, and third sessions of URS were 86.7%, 92%, and 100%, respectively. Rates of stone retropulsion into the kidneys in the SWL and URS groups were 0% and 6.7%, respectively (P=0.000).

Conclusion

Compared with SWL, URS had significantly higher stone-free rates in patients with proximal ureteral stones. Treatment costs and hospital stay were lower in the SWL group, whereas complication rates were comparable.

## Introduction

Urolithiasis is the most common worldwide cause of morbidity in patients with diseases of the urinary tract [1]. Minimally invasive procedures have eased the removal of urinary tract stones [2]. Although ureteral stones were previously managed by open ureter lithotomy, newer techniques, including shock wave lithotripsy (SWL), refinement of semirigid ureter scopes, flexible ureterorenoscopy, and certain laparoscopic procedures have been shown safe and effective in the treatment of ureteral stones, in adults and children [3-5].

SWL is a minimally invasive technique that can be performed on an outpatient basis for the treatment of proximal ureteral calculi [6]. SWL has adverse effects, including long treatment time, a high percentage of patients requiring retreatment, and lack of patient compliance [7].

Ureteroscopic pneumatic lithotripsy (URS) treatment is another technique increasingly used to remove ureteral calculi, especially distal calculi [8]. The success rate of semirigid URS was shown to be higher for proximal than for distal stones [9]. Because URS is associated with a higher success rate after a single session and a lower rate of multiple visits, URS is preferred to SWL in areas in which healthcare facilities are limited [10]. The present study compared the outcomes of URS and SWL in the management of proximal ureteral calculi.

## Materials and methods

In this trial, 150 patients presenting with proximal ureteral stones at the Department of Urology of Nishter Hospital Multan from November 2018 to November 2019 were allocated 1:1 to undergo URS or SWL. After patients were counseled and informed of the advantages and disadvantages of both procedures, patients were allowed to undergo SWL or URS. All patients provided written informed consent.

Patients were diagnosed clinically, based on history, physical examination and X-rays, ultrasound, and CT of the kidneys, ureter, and bladder (KUB). Patients aged >18 years with proximal radiopaque calculi <2 cm in diameter were enrolled. Patients with urinary tract infection, a previous history of ureteral stone surgery, or a coagulation disorder were excluded, as were pregnant women.

Before URS, patients were administered prophylactic antibodies by intravenous injection. URS was performed under general anesthesia with a video monitor attached to the cystoscope. After a guidewire was inserted into the ureter, a rigid ureteroscope of 9F/11F was used. Stones were broken with a Swiss pneumatic LithoClast, and stone fragments removed with a Dormia basket. Intravenous administration of prophylactic antibodies was continued until 24 hours after the procedure, following which the patients were switched to oral antibiotics for one week. Folly’s catheter was removed on the first postoperative day. Patients were followed up for three months by X-ray or ultrasound of the KUP. The procedure was considered successful if remnant stone fragments were <4 mm in size.

SWL was performed using a Storz Modulith Electromagnetic lithotripter (Karl Storz Lithotripsy-America Inc, Atlanta, GA). Stones were identified by ultrasound and fluoroscopy. Patients were administered 90 shock waves at energy level 2 for one minute, followed by 200 shocks at level 3 or 4. Patients who experienced pain were administered intravenous injections of nalbuphine. Patients with large residual stones were advised to return for a second or even a third session. Patients were monitored via follow-up for three months by X-ray or ultrasound of the KUP. The procedure was considered successful if remnant stone fragments were smaller than 4 mm.

## Results

Of the 150 patients enrolled in this study, 75 underwent SWL and 75 underwent URS. Gender distribution, mean age, body mass index (BMI), stone size, skin to stone distance, and Hounsfield units (HU) did not differ significantly in the two groups (Table 1). Of the 75 patients in the SWL group, 32 (42.7%) had right-sided and 43 (57.3%) had left-sided stones, comparable to the 31 (41.3%) patients with right-sided stones and 44 (58.7%) with left-sided stones in the URS group. The prevalence of hypertension was significantly lower in the SWL than in the URS group (28.0% vs. 45.3%, P=0.028), whereas the prevalence of diabetes mellitus was similar (41.3% vs. 37.3%, P=0.616).

**Table 1:** Demographic characteristics of patients with proximal ureteral stones treated with shock wave lithotripsy and ureteroscopic pneumatic lithotripsy Results reported as mean±standard deviation or as number (%). BMI, body mass index; SWL, shock wave lithotripsy; URS ureteroscopic pneumatic lithotripsy.

Characteristics	SWL group (n=75)	URS group (n=75)	P-value
Age (years)	41.21±3.15	40.98±3.73	0.689
BMI (kg/m^2^)	23.98±1.87	10.51±1.82	0.484
Skin to stone distance	9.33±1.54	9.34±1.52	0.957
Stone size (cm)	10.51±2.31	24.19±1.92	0.969
Hounsfield units of stones	799.77±14.47	801.17±11.8	0.519
Gender			
Male	45 (60%)	47 (62.7%)	0.737
Female	30 (40%)	28 (37.3%)	
Side			
Right side	32 (42.7%)	31 (41.3%)	0.869
Left side	43 (57.3%)	44 (58.7%)	
Hypertension	21 (28%)	34 (45.3%)	0.028
Diabetes mellitus	31 (41.3%)	28 (37.3%)	0.616

The mean procedure time was significantly shorter (61.61±3.21 vs. 85.01±6.75 minutes, P=0.000), while the mean number of sessions was significantly higher (1.51±0.49 vs. 1.01±0.42, P=0.000) in the SWL group than in the URS group. Stone-free rates after the first, second, and third sessions were 64%, 77.3%, and 94.7%, respectively, in the SWL group, and 86.7%, 02%, and 100%, respectively, in the URS group (Table 2).

**Table 2:** Procedural outcomes of patients with proximal ureteral stones treated with shock wave lithotripsy and ureteroscopic pneumatic lithotripsy Results reported as mean±standard deviation or as number (%). SWL, shock wave lithotripsy; URS ureteroscopic pneumatic lithotripsy.

Characteristics	SWL group (n=75)	URS group (n=75)	P-value
Procedural time (minutes)	61.61±3.21	85.01±6.75	0.000
Number of sessions (procedures)	1.51±0.49	1.01±0.42	0.000
Hospital stay (days)	--	1.38±0.21	--
Stone-free rate after first session	48 (64%)	65 (86.7%)	0.001
Stone-free rate after second session	58 (77.3%)	69 (92%)	0.013
Stone-free rate after third session	71 (94.7%)	75 (100%)	0.000
Stone retropulsion into kidney (URS)	0 (0%)	5 (6.7%)	0.000

The rate of stone retropulsion into the kidneys was significantly lower in the SWL group than in the URS group (0% vs. 6.7%, P=0.000). Complication rates, assessed using the Clavien grading, did not differ in these two groups (Table 3).

**Table 3:** Clavien grading system scores in patients with proximal ureteral stones treated with shock wave lithotripsy and ureteroscopic pneumatic lithotripsy Results reported as number (%). SWL, shock wave lithotripsy; URS ureteroscopic pneumatic lithotripsy.

Clavien grade	SWL group (n=75)	URS goup (n=75)	P-value
0=no complications	59 (78.7%)	60 (80%)	0.840
1=deviation from normal post procedural course without need for intervention	7 (9.3%)	9 (12%)	0.597
2=mild complications needing intervention	1 (1.3%)	1 (1.3%)	1.000
3a=postprocedural complications needing intervention without use of general anesthesia	6 (8%)	5 (6.7%)	0.754
3b=complications needing intervention under general anesthesia	3 (4%)	8 (10.7%)	0.117
4a=life-threatening complication needing intensive care management	4 (5.3%)	1 (1.3%)	0.172
4b= life-threatening complication needing intensive care management	4 (5.3%)	1 (1.3%)	0.172
5=death	2 (2.7%)	1 (1.3%)	0.560

## Discussion

Management of patients with urinary lithiasis includes minimally invasive procedures as well as non-invasive modalities such as SWL, thereby reducing the need for open surgery [11]. SWL has been reported to be optimal for proximal stones of the ureter, with antracarporeal lithotripsy being safe for these patients [12]. URS has also been reported safe, with a success rate of 80% in the removal of proximal ureteral stones and with a higher success rate in patients with smaller than larger stones [13]. Experience and skill are necessary to perform URS [14]. Although both SWL and URS are effective and safe, SWL was found to be more effective.

Aboutaleb et al. concluded in his study that although SWL was shown to be safe for the removal ureteral stones, with a higher stone-free rate than URS, the complication rate is higher and hospital stay longer in patients who underwent SWL [15]. A comparison of URS and SWL in patients with proximal stones found no significant differences between these two modalities.

The success rates of both procedures are reduced in patients with a BMI >30 kg/m^2^ and stones with >500 HU [16]. A second study reported similar results that both techniques are equally effective for stone removal, especially for proximal ureteral stones [17].

A comparison of complications in patients undergoing stone removal reported that all complications in patients who underwent SWL were minor nature, with the most common being loin pain, occurring in 21% of patients [18]. A study comparing URS and SWL for ureteral calculi reported that both had similar complication rates of about 4%, whereas episodic treatment was not possible [19].

## Conclusions

URS had significantly higher stone-free rates in patients with proximal ureteral stones as compared with SWL. Treatment cost and hospital stay were lower in the SWL group, whereas complication rates were comparable.
